# Poisoning

**Published:** 1885-01

**Authors:** 


### POISONING.

In the year 1881 there were 569 deaths recorded in England alone from poisoning, while the year 1882 shows a record considerably in excess of this, viz., 599, or one in every 863 of the total deaths registered. Fully two-fifths of these cases are classified under the heading "Accident and Negligence;" the remainder are suicides; and as it is not too much to assume that in nearly every instance such cases are preventable, we purpose calling attention to some of the more common causes of these fatalities, in the hope that the suggestions and warnings thrown out may not be without their influence in producing more care in the handling and use of these dangerous substances. Glancing over the various poisons, we find that the well-known preparations of opium, laudanum, and morphia—opium itself being included—head the list, having caused 85 deaths through accident or negligence. This might have been expected from preparations so largely used in domestic remedies; but the 78 deaths from lead poisoning which follow do surprise us, in view of the fact that the conditions which produce as well as the conditions which mitigate or counteract the effects of this subtle poison are now so well known. Lead is followed by the four stronger acids—hydrochloric, nitric, sulphuric, and carbolic—which among them have caused 34 deaths under the same category. Arsenic, again, caused 9; phosphorus, 11; chlorodyne, 6; chloral, 14; chloroform, 4; soothing syrup, 4, with a host of casualties from substances of minor importance. Reading between the lines of the Registrar-General's report, which it is not difficult to do with the help of the medical journals, we will find that there are two prolific causes of these accidents—first, the giving or taking of overdoses of certain remedies containing poison, and, second, the substitution of one bottle or substance for another, as, for example, where a number of substances are congregated together, as in the case of the domestic cupboard. In the first class may be instanced the giving of overdoses of opiates or soothing preparations to children; the taking of overdoses of narcotics or soothing compounds, such as chloral, by habitual drinkers, and the general familiarity which the handling or using of these powerful agents frequently begets in those habitually using them. In the second class may be instanced such mistakes as the substituting of one bottle containing, say, a poisonous liniment for a mixture intended for internal administration; the hasty and foolish practice of quaffing off a draught from any jug, bottle, or dish without examining the contents. and, lastly, mistakes caused from accumulating within easy access powerful medicines in the hope that they may come of future use.—*Chambers Journal.*

